# DO MORE EMPATHIC OLDER ADULTS RUMINATE MORE? EMOTION REGULATION MATTERS

**DOI:** 10.1093/geroni/igac059.1026

**Published:** 2022-12-20

**Authors:** Meng Huo, Kate Leger, Kira Birditt, Karen Fingerman

**Affiliations:** University of California, Davis, DAVIS, California, United States; The University of Kentucky, Lexington, Kentucky, United States; University of Michigan, Ann Arbor, Michigan, United States; The University of Texas at Austin, Austin, Texas, United States

## Abstract

Older adults differ in their responses to distress, and those who tend to ruminate report poor health. We sought to examine whether trait empathy, the tendency to share and understand others’ distress, underlies rumination, and whether this association varies by emotion regulation. Participants included 289 adults aged 65+ in the Daily Experiences and Well-being Study. They reported demographics, empathy, general preferences for emotion regulation strategies, and affect throughout the day as well as daily rumination. Empathy was associated with greater rumination, which was particularly evident in older adults who preferred avoidant strategies. We also found that the link between empathy and rumination was attenuated on days when older adults had lower negative affect variability. This study identifies empathy as a key factor that underlies individual differences in rumination, a key precursor of psychopathologies, and also suggests emotion regulation as a promising target of interventions that can promote older adults’ health.

